# Diagnostic yield of colonoscopy surveillance in testicular cancer survivors treated with platinum-based chemotherapy: study protocol of a prospective cross-sectional cohort study

**DOI:** 10.1186/s12876-021-01639-2

**Published:** 2021-02-12

**Authors:** Berbel L. M. Ykema, Tanya M. Bisseling, Manon C. W. Spaander, Leon M. G. Moons, Dorien van der Biessen-van Beek, Lisette Saveur, Martijn Kerst, Sasja F. Mulder, Ronald de Wit, Danielle Zweers, Gerrit A. Meijer, Jos H. Beijnen, Iris Lansdorp-Vogelaar, Flora E. van Leeuwen, Petur Snaebjornsson, Monique E. van Leerdam

**Affiliations:** 1 Department of Gastroenterology and Hepatology, Netherlands Cancer Institute, Plesmanlaan 121, 1066 CX Amsterdam, The Netherlands; 2 Department of Gastroenterology and Hepatology, Radboud University Medical Center, Nijmegen, The Netherlands; 3 Department of Gastroenterology and Hepatology, Erasmus University Medical Center, Rotterdam, The Netherlands; 4 Department of Gastroenterology and Hepatology, University Medical Center Utrecht, Utrecht, The Netherlands; 5 Department of Medical Oncology, Netherlands Cancer Institute, Amsterdam, The Netherlands; 6 Department of Medical Oncology, Radboud University Medical Center, Nijmegen, The Netherlands; 7 Department of Medical Oncology, Erasmus University Medical Center, Rotterdam, The Netherlands; 8 Department of Medical Oncology, University Medical Center Utrecht, Utrecht, The Netherlands; 9 Department of Pathology, Netherlands Cancer Institute, Amsterdam, The Netherlands; 10 Department of Pharmacy and Pharmacology, Netherlands Cancer Institute, Amsterdam, The Netherlands; 11 Department of Public Health, Erasmus University Medical Center, Rotterdam, The Netherlands; 12 Department of Epidemiology, Netherlands Cancer Institute, Amsterdam, The Netherlands; 13 Department of Gastroenterology and Hepatology, Leiden University Medical Center, Leiden, The Netherlands

**Keywords:** Colorectal cancer, Colorectal neoplasia, Colonoscopy, Surveillance, Testicular cancer, Platinum-based chemotherapy, Surveillance, Carcinogenesis

## Abstract

**Background:**

Testicular cancer (TC) survivors have an increased risk of various second primary malignancies. A recent cohort study detected an increased risk of colorectal cancer (CRC) in TC survivors treated with platinum-based chemotherapy with a hazard ratio of 3.9. CRC risk increased with higher cisplatin-dose. We know that colonoscopy surveillance in high-risk populations results in reduced incidence and mortality of CRC. TC survivors treated with platinum-based chemotherapy can potentially benefit from colonoscopy surveillance; however, to which extent is unknown. Furthermore, the pathogenesis of these secondary CRCs is unknown, and better insights into the carcinogenesis may affect surveillance decisions.

**Methods:**

This prospective multicenter study will be performed in four Dutch hospitals. TC survivors are eligible if treated with ≥ 3 cycles of cisplatin before age 50. Colonoscopy will be performed ≥ 8 years after initial treatment (minimum and maximum ages at colonoscopy, 35 and 75 years, respectively). The primary aim of the study is the diagnostic yield of advanced neoplasia detected during colonoscopy. As secondary aim, we will evaluate the molecular profile of advanced colorectal neoplasia and will assess current platinum levels in blood and urine and correlate blood-platinum levels with prevalence of colorectal lesions. Furthermore, we will investigate effectiveness of fecal immunochemical testing (FIT) and burden of colonoscopy by two questionnaires. Demographic data, previous history, results of colonoscopy, hemoglobin level of FIT and results of molecular and platinum levels will be obtained. Yield of colonoscopy will be determined by detection rate of adenoma and serrated lesions, advanced adenoma detection rate and CRC detection rate. The MISCAN model will be used for cost-effectiveness analyses of CRC surveillance. With 234 participants undergoing colonoscopy, we can detect an absolute difference of 6% of advanced neoplasia with 80% power.

**Discussion:**

TC survivors treated with cisplatin-based chemotherapy can benefit from CRC surveillance. Evaluation of the diagnostic performance and patient acceptance of CRC surveillance is of importance to develop surveillance recommendations. Insight into the carcinogenesis of cisplatin-related advanced colorectal lesions will contribute to CRC prevention in the increasing number of TC survivors. The results may also be important for the many other cancer survivors treated with platinum-based chemotherapy.

***Trial registration*:**

Clinical Trials: NCT04180033, November 27, 2019, https://clinicaltrials.gov/ct2/show/NCT04180033.

## Background

Due to improved treatment strategies, testicular cancer (TC) survival has strongly increased over the past decades, especially since the introduction of cisplatin-based chemotherapy [1]. As TC usually is diagnosed at a young age, this high cure rate results in a long life-expectancy [2–5]. Yet, this life-expectancy can be compromised by late adverse treatment effects such as second primary malignancies [6].

A cohort study detected an increased risk of developing colorectal cancer (CRC) in TC survivors treated with platinum-based chemotherapy with a hazard ratio of 3.9 compared with the general population [7]. A dose–response relationship between platinum and gastro-intestinal second primary malignancies including CRC has been demonstrated [7]. An increased risk for gastro-intestinal cancers, mainly CRC, pancreatic and gastric cancer, has also been described in childhood cancer survivors treated with platinum-based chemotherapy [8]. Other studies have also reported an increased incidence of CRC following TC treatment with a RR varying between 1.3 and 2.3 compared with the general population [9–15]. The risk of developing CRC remained significantly elevated for a long period after TC treatment with elevated risks reported even after 35 years [12, 13, 16]. Therefore we hypothesize that comparable to other high-risk groups for developing CRC, colonoscopy surveillance may be beneficial in the follow-up of TC survivors [17, 18]. Because colonoscopy is an invasive procedure, surveillance by fecal immunochemical testing (FIT) may be a good alternative. FIT is effective and a non-invasive CRC screening strategy, however, effectiveness in high-risk groups is not known.

Currently, the pathogenesis of CRC in TC survivors exposed to cisplatin is poorly understood. In several studies, platinum was still detectable in serum and urine up to 20 years after treatment [19–21]. Cisplatin can cause DNA damage by inducing crosslinks. Therefore, CRCs and their precursor lesions in TC survivors may differ histopathologically or molecularly from sporadic CRC.

We designed a prospective study to determine the diagnostic yield of advanced neoplasia (advanced adenomas, advanced serrated lesions or CRC) in TC survivors treated with platinum-based chemotherapy as these patients have an increased risk for developing CRC. FIT surveillance is non-invasive and may be a good alternative to colonoscopy surveillance. The accuracy of FIT with regard to the detection of advanced neoplasia will be determined. Furthermore, the burden of colonoscopy and cost-effectiveness of different CRC surveillance strategies will be evaluated. Additionally, the level of platinum in plasma and urine and the histopathological and molecular pattern of advanced neoplasia will be assessed. Insight into the pathogenesis and the association with platinum levels may result in a tailored CRC surveillance program for TC survivors.

## Methods/design

### Objectives

The primary objective of this study is to evaluate the diagnostic yield of advanced colorectal neoplasia by surveillance colonoscopy in TC survivors treated with platinum-based chemotherapy. Advanced neoplasia is defined as advanced adenoma (high-grade dysplasia, > 25% villous component or ≥ 10 mm), advanced serrated lesion (dysplasia or ≥ 10 mm diameter) or CRC [22, 23].

The secondary objectives are to assess the effectiveness of FIT in detecting advanced neoplasia and to examine patient perception (burden, acceptance and satisfaction). We will evaluate which CRC surveillance strategy (FIT or colonoscopy) is most cost-effective in TC survivors. Furthermore, we will evaluate the clinicopathological and molecular characteristics of advanced neoplasia and relate that to platinum level in plasma and urine in TC survivors.

This prospective cross-sectional cohort study was approved by the Medical Ethics Committee of the Netherlands Cancer Institute (Dutch Trial Registry (NL68513.031.19) and registered at Clinical Trials (NCT04180033)) and is currently ongoing.

### Study design

#### Population

TC survivors will be selected from a well-defined multicentre cohort including 5.848 1-year TC survivors who were treated between 1976 and 2007 [7]. Inclusion criteria for this study are as follows: (1) treatment of primary TC should have consisted of ≥ 3 cycles of platinum-based chemotherapy consisting of cisplatin; (2) diagnosis of TC was made before the age of 50 years; (3) initial treatment for TC administered at least 8 years prior to inclusion; (4) the patients should be at least 35 years of age and not older than 75 years of age and (5) detection and potential treatment of advanced colorectal neoplasia should be considered beneficial, i.e. life-expectancy of at least 5 years.

TC survivors are not eligible for participation in this study if they meet one of the following exclusion criteria: a history of proctocolectomy, colonoscopy surveillance for other indications (including hereditary CRC syndrome, familial CRC syndrome, inflammatory bowel disease, history of colorectal adenoma or CRC), colonoscopy in the past 3 years, ongoing cytotoxic treatment or radiotherapy for malignant disease, coagulopathy (prothrombin time < 50% of control; partial tromboplastin time > 50 s) or anticoagulants (fenprocoumon, acenocoumarol, or direct oral anticoagulants) or platelet aggregation inhibitors that cannot be stopped or safely bridged if necessary, comorbidity leading to an impaired physical performance (World health organization (WHO) performance status 3–4) or mental retardation, limited Dutch language skills or no informed consent.

**Fig. 1:**
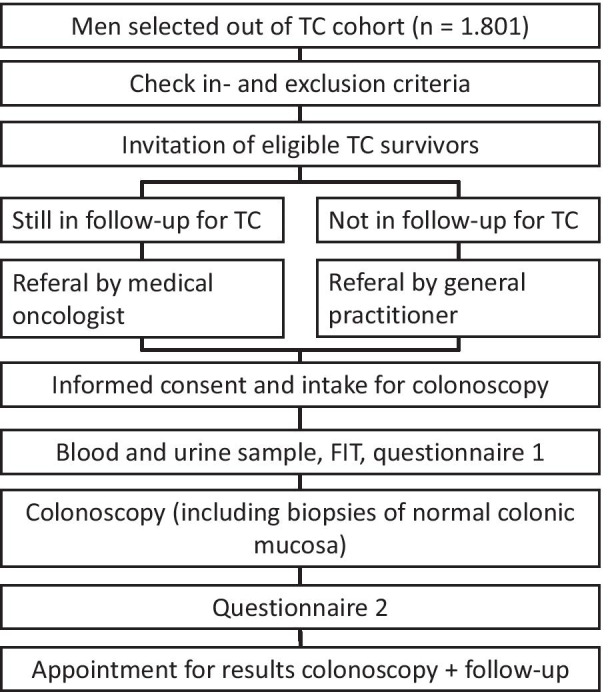
Flowchart of study. Abbreviations: *FIT* Fecal immunochemical test; *TC* testicular cancer

A total of 1.801 men treated in the four different hospitals in the Netherlands could potentially be eligible. Individuals who do meet the inclusion criteria will be invited for participation in one of the four Dutch study centers (Netherlands Cancer Institute, Amsterdam, Erasmus University Medical Center, Rotterdam, Radboud University Medical Center, Nijmegen and University Medical Center Utrecht, Utrecht). The flowchart of the design of this study is shown in Fig. 1. Participants who are eligible for participation will be invited for this study. Referral to the gastroenterology department will occur via the general practitioner or medical oncologist, depending whether the individual is still in follow-up for TC. Informed consent will be concluded by a gastroenterologist or nurse specialist, when the participant was informed adequately.

For patients who will be excluded due to colonoscopy in the 3 years prior to invitation we will evaluate the yield of colonoscopy retrospectively. This study was approved by the Institutional Review Board of the Netherlands Cancer Institute (IRBd19-236).

#### Control population

The diagnostic yield of colonoscopy in TC survivors will be compared to the NordICC study. This is a European randomized trial for population-based primary colonoscopy screening for colorectal cancer. Participants were between 55 and 64 years of age and we selected only male participants to compare with our male cohort of TC survivors [24]. For molecular profiling, data will be compared with existing molecular data of colorectal lesions (mainly polyps) diagnosed in Dutch patients before the age of 70 years [25].

#### Sample size calculation

The power calculation is based on the primary endpoint of this study: the diagnostic yield of advanced colorectal neoplasia by screening colonoscopy. The prevalence of advanced colorectal neoplasia in the NordICC study population was 10.3% among the 6,493 male participants who underwent colonoscopy screening (based on the most recent data) [24]. We will use the data from the male participants of the NordICC study aged 55–64 years old and use frequency matching with 5-year strata of the NorcICC male patients to patients in our study. We do believe that colonoscopy surveillance is justified with an increase detection rate of advanced neoplasia adjusted for age of 6%. In order to detect this 6% difference in advanced neoplasia in TC survivors with at least 80% power (using a two-sided alpha of 5%), we need to include at least 234 TC survivors. A margin of 40% will be considered for non-responders and drop-outs based on previous participation rates and therefore 338 men will be approached. An interim analysis will be performed after the inclusion of 100 participants.

### Study procedures

#### Colonoscopy

The bowel preparation will be performed as for routine colonoscopy by ingesting a commonly prescribed oral electrolyte lavage solution. Conscious sedation (with midazolam and/or fentanyl citrate or propofol) and cardiopulmonary monitoring will be used according to standard protocol. The colonoscopy will be performed by experienced gastroenterologists having performed at least 1000 colonoscopies or under direct supervision of an experienced gastroenterologist. When colorectal neoplasia is detected, polypectomy will be performed or biopsies will be obtained according to standard protocol. In case of a polyp ≥ 10 mm, four field biopsies will be taken around the polyp. Additional fresh frozen material of non-neoplastic mucosa will be taken for study purposes from all participants to perform additional molecular analyses (see section histology, immunohistochemistry and molecular pathology). Four biopsies of normal mucosa (fresh frozen) of the transverse colon and two to four biopsies of normal mucosa of the descending colon (fresh frozen) will be taken.

#### Histology, immunochemistry and molecular pathology

An experienced gastro-intestinal pathologist will perform routine histopathological evaluation of colorectal neoplasia. All advanced neoplasia will be requested from all four hospitals and will be re-evaluated by the same pathologist (PS) and.immunohistochemical and molecular analysis will be performed on these advanced lesions. Immunohistochemistry (IHC) of the MMR proteins (MLH1/MSH2/MSH6 and PMS2) will be performed and DNA will be isolated. A mutational panel (Sequenom Massarray including AKT1, BRAF, DDR2, EGFR, MEK1, PIK2CA, KRAS and NRAS) will be executed. The definite analyses will be based on an ongoing retrospective study on CRC in TC survivors.

#### Biopsies

Biopsies of normal colorectal tissue from the transverse and descending colon will be obtained after informed consent, as previously described. This fresh frozen tissue will be evaluated to further understand the effect of platinum-based chemotherapy on endoscopically normal mucosa.

#### Questionnaires

The participant will receive a first questionnaire together with the information about the colonoscopy after informed consent is given, before colonoscopy is performed. This questionnaire will evaluate risk factors for CRC risk (including familial history), quality of life, the physical and mental functioning of the patient and the expected burden of colonoscopy. A second questionnaire will be sent to the participant one week after the colonoscopy. This questionnaire will again assess quality of life, the physical and mental functioning of the patient and will determine the experienced burden of colonoscopy. The questionnaires will include information on the expected/experienced burden, embarrassment and pain of the bowel preparation and the colonoscopy procedure.

#### Fecal immunochemical test

Patients will receive instructions and the stool test (FIT, FOBgold test) after informed consent. Prior to bowel preparation for the surveillance colonoscopy, patients are asked to collect a stool sample to perform FIT and send the stool kit to the laboratory for processing and analysis. The FIT is a quantitative test and the level of hemoglobin will be detected in µg Hb/g feces. The sensitivity, specificity, positive predictive value, negative predictive value and area under the curve can be calculated for (advanced) neoplasia with the colonoscopy of the participant as a reference.

#### Platinum in plasma and urine

We will measure the platinum level in a single plasma (6 mL EDTA containing tube) and a urine sample using inductively coupled plasma mass spectrometry (ICP-MS). A blood and urine sample will be collected on the day of intake or before the colonoscopy. ICP-MS will be performed according to a protocol previously described [19, 26, 27].

#### Cost-effectiveness analysis

Cost-effectiveness analysis of colonoscopy surveillance will be evaluated using the microsimulation screening analysis (MISCAN) model [28, 29]. In this model, the influence of the implementation of a surveillance program (colonoscopy or FIT) will be simulated. This model can estimate the incidence, prevalence and mortality of CRC, and the results and effects of surveillance based on the prevalence of advanced neoplasia detected during this current study and taking to account the mortality risk of TC survivors [30]. The costs and number of life-years gained for the population with and without the implementation of surveillance will be evaluated. Based on these results, we can make recommendations for CRC surveillance based on the most cost-effective strategy.

## Discussion

In this prospective study, TC survivors treated with platinum-based chemotherapy will be offered a colonoscopy in order to evaluate the diagnostic yield in detecting advanced neoplasia as we hypothesize that this population benefits from CRC surveillance. This study is currently ongoing and we expect to complete inclusion in June 2022.

Colonoscopy reduces the risk of CRC and CRC related mortality [31–33]. CRC screening programs for the general average-risk population have been implemented in many countries [24, 34]. There are different CRC screening modalities including primary colonoscopy screening and fecal occult blood test screening with colonoscopy in case of a positive test result. For high-risk-groups a more intensified surveillance program is recommended, and the interval used differs between the different high-risk-groups [18, 35]. For individuals with a family history of CRC and an estimated increased risk of CRC of 2.5 compared to the general population, colonoscopy surveillance is advised every 5 years from the age of 45 years [17, 18]. During colonoscopy precursor lesions of CRC can directly be removed which has been shown to reduce CRC incidence and mortality in high-risk-groups [31, 36–39]. TC survivors treated with platinum-based chemotherapy do have a 3.9-fold increased risk. Due to the increased CRC risk, we will evaluate the diagnostic yield of colonoscopy surveillance. The diagnostic yield of advanced neoplasia detected during colonoscopy in TC survivors will be compared to the male population of the NordICC study, which contains males with an average risk for developing CRC [24].

The participation rate of CRC surveillance is of great importance to benefit as many TC survivors as possible. Currently, most TC survivors are unaware of the increased risk of developing CRC. In a Dutch pilot study with primary colonoscopy screening the participation rate of colonoscopy was only 21% [34]. On the other hand, colonoscopy participation in a high risk group of familial risk was reported between 40% [40]. Therefore, FIT surveillance should be considered, as FIT is less invasive than colonoscopy and therefore could increase the participation rate in CRC surveillance. There are already studies showing that FIT surveillance may be beneficial in different risk groups for CRC by choosing a low cut-off [41]. However, in familial colorectal cancer and other patients with an increased risk of CRC, the accuracy of FIT is unknown.

In high-risk-groups for developing CRC, colonoscopy surveillance has been shown to be cost-effective [42, 43]. The MISCAN model will be adapted by specific CRC risk data and competing mortality risk data of TC survivors. We will than evaluate the CEA of both colonoscopy and FIT screening at different cut-off levels. This will result in an advise for further surveillance in TC survivors.

We hypothesize that the carcinogenesis of (the precursor lesions of) CRC in TC survivors treated with platinum-based chemotherapy differs from sporadic CRC. It is known that cisplatin can induce cytotoxicity through the formation of DNA crosslinks due to covalent bonds between purines [44–51]. The analysis which will be performed on the tissue obtained from this study will depend on an ongoing retrospective study where the histopathological and molecular characteristics of CRC diagnosed in TC survivors treated with platinum-based chemotherapy will be evaluated (data not yet published).

Finally, level of platinum in plasma will be determined in order to see if there is correlation between the level of platinum in plasma and the presence of (advanced) neoplasia detected during the colonoscopy. It is unknown whether persisting retention of platinum adducts in plasma correlate with the increased risk of advanced neoplasia [19, 20]. Furthermore, urine platinum level will be evaluated as cisplatin is renally excreted, and was also detected 17 years after platinum treatment [7]. Maybe platinum levels in plasma and urine may guide indication for colonoscopy surveillance.

This protocol describes a prospective cross-sectional cohort study where we will evaluate the diagnostic yield of advanced neoplasia in TC survivors treated with platinum-based chemotherapy. Furthermore, we will evaluate the effectiveness of FIT, patient acceptance and burden of colonoscopy and also the most cost-effective CRC surveillance strategy. Additionally, we aim to gain insights into the pathogenesis of CRC in TC survivors which includes determining platinum levels in plasma and urine. Through this study we aim to provide CRC surveillance recommendations. This knowledge may also be of guidance for other cancer survivors who have received platinum-based chemotherapy for other types of malignancies.

## Data Availability

The datasets generated and/or analysed during this study are not publicly available due to individual privacy but are available from the corresponding author on reasonable request.

## Abbreviations

CRC: Colorectal cancer
FIT: Fecal immunochemical test
MISCAN: MIcrosimulation screening analysis
MMR: Mismactch repair
TC: Testicular cancer
